# Enhanced Expression of CD47 Is Associated With Off-Target Resistance to Tyrosine Kinase Inhibitor Gefitinib in NSCLC

**DOI:** 10.3389/fimmu.2019.03135

**Published:** 2020-01-31

**Authors:** Annunziata Nigro, Luca Ricciardi, Ilaria Salvato, Francesco Sabbatino, Monica Vitale, Maria Assunta Crescenzi, Barbara Montico, Massimo Triggiani, Stefano Pepe, Cristiana Stellato, Vincenzo Casolaro, Jessica Dal Col

**Affiliations:** ^1^Department of Medicine, Surgery and Dentistry “Scuola Medica Salernitana,” University of Salerno, Baronissi, Italy; ^2^Immunopathology and Cancer Biomarkers, Translational Unit, Centro di Riferimento Oncologico di Aviano (CRO), IRCCS, Aviano, Italy

**Keywords:** CD47, non-small cell lung cancer, tyrosine kinase inhibitor resistance, phagocytosis checkpoint, innate immunity, cancer immune surveillance

## Abstract

Mutual interactions between cancer cells and the tumor microenvironment importantly contribute to the development of tyrosine kinase inhibitor (TKI) resistance in patients affected by EGFR-mutant NSCLC. In particular, immune recognition-associated proteins with impact on tumor cell clearance through phagocytosis, such as CD47 and calreticulin, could contribute to adaptive resistance and immune escape. Preclinical studies targeting the anti-phagocytic CD47 molecule showed promising results in different cancer types including lung cancer, but no data are available on its role in patients acquiring resistance to EGFR TKI treatment. We analyzed the functional contribution of CD47 and calreticulin to immune surveillance and evasion in a panel of NSCLC cell lines carrying sensitizing or resistant mutations in the EGFR gene, following treatment with the TKI gefitinib and after *in vitro* development of gefitinib resistance. While CD47 and calreticulin protein levels were markedly variable in both EGFR-mutant and wild-type cell lines, analysis of NSCLC transcriptomic dataset revealed selective overexpression of CD47 in patients carrying EGFR mutations. EGFR inhibition significantly reduced CD47 expression on the surface of pre-apoptotic cells, favoring more efficient engulfment of cancer cells by monocyte-derived dendritic cells. This was not necessarily associated with augmented surface exposure of calreticulin or other molecular markers of immunogenic cell death. Moreover, CD47 expression became up-regulated following *in vitro* drug resistance development, and blocking of this protein by a specific monoclonal antibody increased the clearance of EGFR-TKI resistant cells by phagocytes. Our study supports CD47 neutralization by specific monoclonal antibody as a promising immunotherapeutic option for naïve and resistant EGFR-mutant NSCLCs.

## Introduction

Non-small-cell lung cancer (NSCLC) accounts for 85% of all diagnosed cases of lung cancers, with the diagnosis taking place often when the disease is at locally advanced or metastatic stage (1, 2). The discovery of oncogenic driver mutations in almost two-thirds of NSCLCs and the introduction of targeted therapies undeniably improved the outcome in patients with oncogene-addicted adenocarcinoma. However, clinical response to treatment is generally temporary and incomplete (3). Recent studies on the molecular determinants of resistance to tyrosine kinase inhibitors (TKIs) identified two major mechanisms either affecting targeted oncogenes (“on-target” resistance) or molecules residing downstream or belonging to parallel and other pathways (“off-target” resistance) (4). In addition, dynamic interactions between tumor cells and the surrounding microenvironment critically contribute to modifying the response to TKI therapy and influence the development of resistant phenotypes (5–7). It has been demonstrated that in epidermal growth factor receptor (EGFR)-driven lung tumors, anti-tumor immunity is inhibited by activation of the Programmed death 1 (PD-1)/Programmed death ligand 1 (PD-L1) pathway, leading to suppression of effector T cell function and increased levels of pro-inflammatory cytokines (8). As recently shown, PD-L1 expression in tumor cells adversely affects EGFR-TKI efficacy, especially in NSCLC patients with *de novo* resistance (9). Furthermore, the secretion from stromal cells of paracrine factors such as interleukin-6 (IL-6), transforming growth factor-β (TGF-β), and hepatocyte growth factor (HGF) promotes MAP-kinase activation and further supports EGFR TKI resistance development by eluding EGFR pathway inhibition (10).

Immune checkpoint inhibitors (ICIs) targeting the PD-L1/PD-1 axis have been recently approved for the treatment of EGFR- and Anaplastic lymphoma kinase (ALK)-positive NSCL tumors after failure of appropriate targeted therapy (11, 12). While the association of EGFR mutations with high PD-L1 expression suggests the potential efficacy for PD-L1 inhibitors in this setting, treatment with ICIs showed limited efficacy in different cohorts of patients previously treated with an EGFR TKI (13–16) and the outcome did not show correlation with the EGFR mutation subtype. The poor response to ICIs in EGFR-mutated, TKI-resistant patients suggests that other immune-escape mechanisms are at stake in this clinical phenotype.

No studies to date have examined the effects of EGFR TKIs on immune recognition-associated molecules, such as CD47 and calreticulin (CRT), recently found to affect innate immune surveillance. CD47, originally identified as integrin-associated protein (IAP), is a cell-surface immunoglobulin-like molecule that serves as a “don't eat me” signal via its interaction with signal regulatory protein alpha (SIRPα) on phagocytes (17, 18). Loss of CD47 is permissive to homeostatic phagocytosis of aged or damaged cells (19, 20). While CD47 is ubiquitously expressed at low levels on normal cells, multiple hematologic and solid tumors have been found to express higher levels of CD47 compared to their non-transformed counterparts (21–24). Enhanced expression of CD47 has also been reported in primary NSCLC tumors and cell lines (25). Up-regulation of CD47 expression in human cancers negatively regulates anti-tumor immunity through suppression of phagocytosis, and it has been associated with tumor growth and dissemination (18, 25–28). Conversely, CRT is a highly conserved endoplasmic reticulum chaperone protein, which, upon translocation from the endoplasmic reticulum to the cell surface, provides an “eat-me” signal and facilitates capture by macrophages and dendritic cell precursors of cancer cells undergoing immunogenic cell death (ICD) or other stress conditions (29, 30). Fucikova et al. demonstrated that the expression of CRT in NSCLC correlates with increased accumulation of antitumor immune cells and favorable prognosis (31). Given the emerging critical roles of CD47 and CRT in NSCLC adenocarcinomas, in the present study, we assessed whether the EGFR TKI gefitinib modulates their expression in different EGFR-mutated NSCLCs. Furthermore, we analyzed in these cells the functional contribution of these proteins to immune surveillance, while their potential role in surveillance evasion was tested in subsets of NSCLC cell lines rendered TKI resistant *in vitro*.

## Materials and Methods

### Cell Lines

Human NSCLC cell lines NCI-H1975, NCI-H1299, NCI-H1437, and NCI-H1573 were purchased from ATCC (American Type Culture Collection, Manassas, Virginia). PC9 and HCC827 cell lines were obtained from Cell Biology and Biotherapy Unit, Istituto Nazionale Tumori di Napoli, IRCCS “G. Pascale.” The EGFR TKI-resistant cell lines, PC9GR and HCC827GR, were generated by culturing the respective parental cell lines in the presence of increasing concentrations of TKI Gefitinib (from 0.05 to 0.5 μM and from 0.1 to 1 μM, respectively) for 8 weeks, to reach a concentration 10 times higher than the initial IC_50_. Cell lines were cultured in RPMI-1640 medium (Gibco) supplemented with 10% fetal bovine serum (Gibco), 1% L-glutamine (2 mM, Lonza), 1% streptomycin-penicillin (EuroClone) at 37°C in a 5% CO_2_ humidified atmosphere. Short tandem repeat (STR) loci for cell lines authentication were evaluated for all cell lines, by using the GenePrint10-System (Promega).

### Antibodies and Reagents

Anti-CD47-APC, anti-CD47-FITC, anti-CD11c-APC-Vio770, anti-CD11-PE, anti-CD14-FITC, anti-CD80-PE, anti CD83-PE, anti-CD86-PE, anti-HLA-DR-FITC, anti-HLA-ABC-FITC, anti-Hsp70-FITC, and 7-Amino-Actinomycin D (7-AAD) fluorescent DNA dye were purchased from Miltenyi. All monoclonal antibodies (mAbs) used in flow cytometry experiments were used at 1:200 titer unless otherwise specified. Anti-calreticulin-FITC mAb (EPR3924; 1:50) and anti-GAPDH were from Abcam. Anti-mouse/human/rat CD47 mAb or mouse IgG isotype control were purchased from Bio X Cell. Anti-phospho-EGFR (Y1608), anti-EGFR, and anti-phospho-Akt (S473) were from Cell Signaling Technology. Secondary antibodies anti-rabbit IgG-HRP or anti-mouse IgG+IgM+IgA-HRP were from Bethyl Laboratories. Gefitinib was purchased from Selleckchem, and its IC_50_ was determined for each cell line using Cell-Counting Kt-8 (Dojindo Laboratories) according to the manufacturer's instructions. Gefitinib IC_50_ for each cell line, reported in Table 1, was used for all the experiments. Recombinant human granulocyte-macrophage colony-stimulating factor (GM-CSF) was purchased from Miltenyi, and interferon (IFN)-α (IntronA) from SP Europe.

**Table 1:** Histological and mutational characteristics of the six NSCLC cell lines included in the study.

**Cell line**	**Histology**	**Tumor source**	**Mutant gene**	**EGFR mutation**	**Mutation effect on EGFR function**	**Gefitinib sensitivity**
PC9	NSCLC adenocarcinoma	Primary	EGFR	E746-A750 deletion	Activating, biomarker for TKI therapy	IC_50_ = 0.05 μM
HCC827	NSCLC adenocarcinoma	Primary	EGFR	E746-A750 deletion	Activating, biomarker for TKI therapy	IC_50_ = 0.1 μM
H1975	NSCLC adenocarcinoma	Primary	EGFR TP53 PIK3CA	L858R T790M	Activating, biomarker for TKI therapy Secondary mutation associated with TKI acquired resistance	IC_50_ = 15 μM
H1299	NSCLC large-cell carcinoma	Metastasis, lymph node	NRAS TP53	WT		IC_50_ > 15 μM
H1437	NSCLC adenocarcinoma	Metastasis, pleural effusion	CDKN2A TP53	WT		IC_50_ > 15 μM
H1573	NSCLC adenocarcinoma	Metastasis, soft tissue	KRAS TP53 PIK3CA	WT		IC_50_ > 15 μM

*The indicated gefitinib IC_50_ values were measured experimentally*.

### Protein Extraction and Western Blot

For total protein extraction, cells were directly lysed in a buffer containing 50 mM Tris/HCl at pH 7.5, 150 mM NaCl, 2 mM EDTA, 2 mM EGTA, 25 mM NaF, 25 mM β-glycerolphosphate, 0.1 mM Na_3_VO_4_, 0.1 mM PMSF, 0.2% Triton X-100, 0.3% Nonidet P40, and a cocktail of protease inhibitors (100 X, EuroClone). After incubation on ice for 30 min, the lysate was centrifuged at 13,000 rpm for 15 min at 4°C. A total of 15 μg of protein per well was separated by 4–15% sodium dodecyl sulfate-polyacrylamide gel electrophoresis and transferred to nitrocellulose membranes. Membranes were then blocked with 5% milk-TBST buffer (TBS plus 0.1% Tween-20) for 1 h at room temperature and incubated with primary antibodies overnight at 4°C, washed with TBST buffer three times, and incubated with corresponding secondary antibodies at room temperature for 45 min. Signals were detected by the “Pierce ECL Western Blotting Substrate” method (Thermo Fisher Scientific) and analyzed by the ImageLab software, using the Chemidoc image acquisition and analysis tool (BioRad).

### Flow Cytometry Analysis

To measure the cell surface expression of CD47 and CRT, 2.5 × 10^5^ cells were seeded in 6-well plates and treated with gefitinib at their specific IC_50_ (see Table 1) or corresponding proportions of DMSO solvent for 48 h. Cells were stained with primary antibodies for 20 min at room temperature (RT). Seven-AAD was added to exclude non-viable cells from the analyses. For intracellular labeling with anti-CD47-FITC mAb, cells were fixed with 4% paraformaldehyde (PFA) and permeabilized with 100% cold methanol. Cells were acquired using a BD FACSVerse™ flow cytometer (BD Biosciences) and the data were analyzed using the BD FACSuite™ software.

### Differentiation of Dendritic Cells From CD14^+^ Monocytes Isolated From Peripheral Blood

Human peripheral blood mononuclear cells (PBMCs) were isolated from whole blood donated by healthy volunteers using Ficoll HyPaque (GE Healthcare). Monocytes were isolated from PBMCs by positive selection onto MACS LS columns using CD14 MicroBeads (Miltenyi) following the manufacturer's protocol. The resulting cell suspensions, containing at least 85% monocytes, were seeded in 6-well plates at a concentration of 2.0 × 10^6^ cells/ml and cultured for 3 days at 37°C in a 5% CO_2_ humidified atmosphere in CellGenixTM GMP DC medium (CellGenix GmbH) supplemented with 1% streptomycin-penicillin (EuroClone), and in the presence of 500 ng/ml GM-CSF and 10,000 U/ml IFN-α (32). Differentiation of dendritic cells was confirmed by immune phenotype analysis for the established dendritic cell markers: CD80, CD83, CD86, CD11c, and HLA-DR.

### *In vitro* Phagocytosis Assay

Dendritic cells were plated in 24-well plates (10^5^ cells/well). After 48 h, lung tumor cells treated with gefitinib at their specific IC_50_ (see Table 1) or DMSO carrier were labeled with DiO cell-labeling solution (Vybrant Cell-Labeling Solution, Molecular Probes) and added to dendritic cells at a 1:1 ratio. Where indicated, tumor cells were incubated with anti-mouse/human/rat CD47 mAb (10 μg/ml, Bio X Cell) or mouse IgG isotype control (10 μg/ml, Bio X Cell) prior to culture with dendritic cells. Following 2.5 h co-culture at 37°C, cells were washed twice with PBS and then labeled with anti-CD11c mAbs (1:200, Miltenyi). Phagocytosis was determined by flow cytometry detection of dendritic cells double positive for CD11c and DiO cell-labeling solution.

### Annexin V/PI Apoptosis Assay

Tumor cells were seeded into 6-well plates (2.5 × 10^5^ cells per well) and cultured (72 h) in the absence or presence of gefitinib at their specific IC_50_ (see Table 1). Apoptosis was assessed by cytofluorimetric analysis using FITC-Annexin V Apoptosis Detection Kit (Dojindo Laboratories) according to the manufacturer's instructions.

### Analysis of CD47 and CRT mRNA in Transcriptomic Databases of NSCL Adenocarcinomas

CD47 and CRT mRNA expression was determined in a publicly available microarray database collecting frozen primary NSCLC specimens with different oncogenic driver mutations (GEO accession number GSE31210) (33, 34). Only the Jetset best probe sets were considered (35), 226016_at for CD47 and 214315_x_at for CRT.

### Statistical Analysis

Protein expression levels of CD47 and CRT in the different cell lines were compared using ANOVA test with Fisher's *post hoc* multiple comparison analysis, whereas mRNA expression levels in different groups of patients were compared using the non-parametric Kruskal-Wallis test. The effect of gefitinib on CD47 and CRT surface expression and on phagocytosis was determined by two-tail paired Student's *t*-test, based on a minimum of three experiments/different donors. The effect of a CD47-neutralizing antibody on phagocytosis was determined using ANOVA test with Fisher's *post hoc* multiple comparison analysis. Statistical analysis was performed using GraphPad Prism 5 (GraphPad Software, Inc. La Jolla, CA, USA).

## Results

### Treatment With Gefitinib Modulates CD47 and CRT Surface Expression in NSCLC Cell Lines

**Figure 1:**
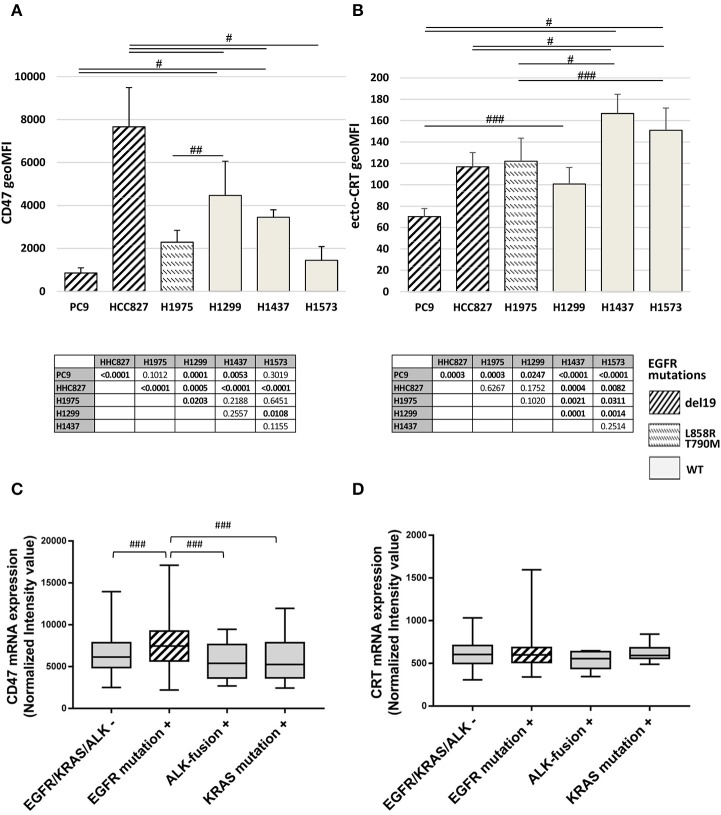
Surface CD47 and CRT expression in EGFR wild-type and mutant NSCLC cells. Surface CD47 **(A)** and ecto-CRT protein expression **(B)** shown as geometric MFI in a panel of six different NSCLC cell lines. Each histogram represents the mean (± SD) of three to five independent experiments. Comparisons made by ANOVA with Fisher's *post hoc* multiple comparison analysis. ^###^*p* < 0.03, ^##^*p* < 0.01, ^#^*p* < 0.0005. Below each histogram, a matrix table where all *p* values resulting from *post hoc* analysis are reported. Expression levels of CD47 **(C)** and CRT mRNA **(D)** in 226 untreated primary NSCL adenocarcinomas (GEO accession number GSE31210). Middle lines in box plots represent the medians and whiskers represent 5–95% CI (^###^*p* < 0.03, Kruskal-Wallis test).

The basal expression levels of CD47 and surface-exposed CRT (ecto-CRT) were investigated in a panel of six NSCLC cell lines harboring different mutations on EGFR as well as on other genes connected or not to the EGFR pathway (Table 1). Flow cytometry analysis showed that expression levels of surface CD47 and ecto-CRT are markedly variable among the different cell lines and independent of the type of EGFR mutations (Figures 1A,B). Retrospective analysis of a published dataset of microarray expression data from frozen primary lung adenocarcinoma specimens allowed us to investigate CD47 and CRT mRNA levels in patients (*N* = 226) with different oncogenic driver mutations (GEO accession number GSE31210) (33, 34). As shown in Figure 1C, patients with EGFR mutations expressed higher levels of CD47 mRNA compared to patients with KRAS mutations, ALK-fusion, or no mutations in these three driver oncogenes (Figure 1C, *p* ≤ 0.03 Kruskal-Wallis test). In contrast, in the same dataset, CRT mRNA expression showed no significant differences among the four groups of patients (Figure 1D).

**Figure 2:**
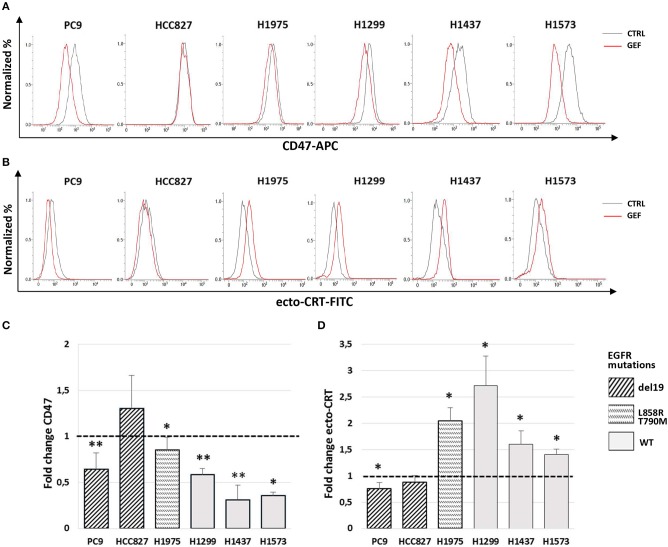
Modulation by gefitinib of surface CD47 and CRT expression in EGFR wild-type and mutant NSCLC cells. Flow cytometric profiles of surface CD47 **(A)** and ecto-CRT expression **(B)** on DMSO-treated (CTRL, gray lines) and gefitinib-treated (GEF, red lines) NSCLC cells. Histograms show the mean (± SD) of fold changes of CD47 **(C)** and ecto-CRT **(D)** geometric MFI, relative to DMSO-treated controls (*N* = 3–5, *p < 0.05, **p < 0.01 paired two-tailed Student's *t*-test).

Next, we assessed the ability of gefitinib to modulate CD47 and CRT surface expression in the cell lines tested. Cells were cultured for 48 h with gefitinib concentrations corresponding to IC_50_ identified for each cell line (listed in Table 1) or an equivalent DMSO amount. Immunoblotting analysis of phospho-EGFR confirmed the inhibition of EGFR activating phosphorylation by gefitinib (Figure S1). Flow cytometry analysis of viable cells showed that gefitinib significantly down-regulated CD47 surface expression in all cell lines, except for HCC827, one of the two cell lines harboring the EGFR exon 19 deletion (del19 mutation) (Figures 2A,C). Concomitantly, gefitinib treatment promoted a significant increase of ecto-CRT expression in all cell lines carrying wild-type EGFR and in H1975, whereas no significant difference was induced in HCC827 and a slow, but consistent ecto-CRT reduction was detected in PC9 cells (Figures 2B,D).

### Gefitinib-Induced Modulation of CD47 and CRT Is Independent on Immunogenic Cell Death Promotion

**Figure 3:**
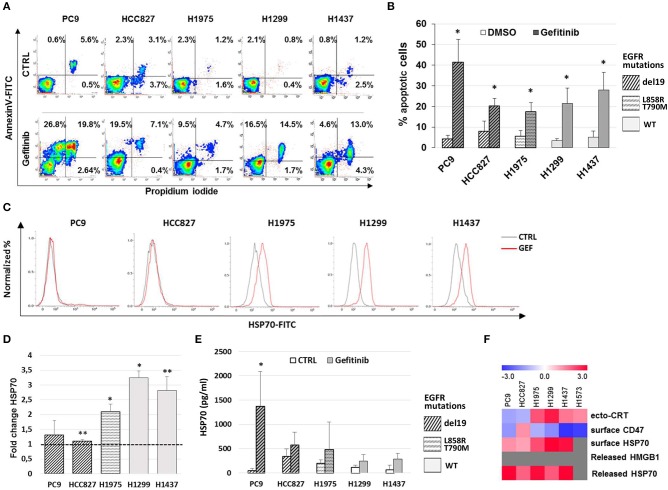
Gefitinib-induced apoptosis in NSCLC cell lines is not immunogenic. **(A)** Representative counter plot of Annexin V/PI assay for gefitinib-treated (72 h) NSCLC cell lines and **(B)** mean (± SD, *N* = 3–6). **(C)** Flow cytometric profiles of surface HSP70 expression on NSCLC cell lines treated for 48 h with DMSO (CTRL, gray lines) or gefitinib (GEF, red lines). **(D)** Mean (± SD, *N* = 3–5) of fold changes (over DMSO-treated controls) of cell surface HSP70 geometric MFIs in gefitinib-treated NSCLC cell lines. **(E)** Mean (± SD, *N* = 3–5) HSP70 concentrations in NSCLC supernatants after gefitinib or DMSO (CTRL) treatment (*p < 0.05, **p < 0.01, paired two-tailed Student's *t*-test). **(F)** The heatmap summarizes the response to gefitinib of established markers of ICD in the tested NSCLC cell lines, with red and blue reporting increased and decreased values over DMSO-treated cells, respectively.

Previous work has shown that the anti-EGFR mAb cetuximab induces immunogenic cell death (ICD) in colon cancer cells by triggering endoplasmic reticulum stress response and CRT translocation, depending on the mutational status of the EGFR signaling pathway (36). Therefore, we next investigated if the effects of gefitinib on CD47 and ecto-CRT expression were associated to the induction of ICD in NSCLC cells. The markers of ICD evaluated together with the ecto-CRT expression were the induction of apoptosis, the surface exposure of CRT heat-shock protein 70 (HSP70), and the release of HSP70 and of high mobility group box-1 (HMGB1) (37). Consistently with the spatiotemporal sequence of ICD markers, surface exposure of proteins was measured in pre-apoptotic cells after 48 h of treatment, whereas the release of HSP70 and HMGB1 was measured 72 h after treatment. As shown in Figures 3A,B, treatment with gefitinib significantly increased the frequency of cells undergoing apoptosis relative to DMSO-treated cells. Moreover, gefitinib increased the surface expression of HSP70 by ≥50% in all cell lines except in cell lines carrying EGFR exon 19 deletion, PC9, and HCC827 (Figures 3C,D), in agreement with findings on ecto-CRT expression in these two cell lines (Figures 2B,D). In contrast, as shown in Figure 3E, gefitinib increased HSP70 release over control-treated cells only in the PC9 cell line. Lastly, gefitinib-induced apoptosis was not associated with HMGB1 release, which was not detected in supernatants (data not shown).

Taken together, these results, summarized in Figure 3F, indicate that while gefitinib-promoted apoptosis is associated with several ICD traits, these are not all detectable in each cell line. Therefore, according to recently published findings (38), the results indicate that EGFR inhibition by gefitinib does not induce ICD in the NSCLC cell lines tested. Moreover, even the concomitant activation of the type I interferon pathway, a well-known important mediator of ICD (39), was insufficient to induce ICD in these experiments. In fact, the addition of IFN-α in these cultures, while further increasing the frequencies of apoptotic cells, did not augment the effects of gefitinib on CD47 and ecto-CRT expression (Figures S2A–C) and was also insufficient to induce HMGB1 release (data not shown).

### Gefitinib-Induced CD47 Down-Regulation Promotes Phagocytosis of Tumor Cells by IFN-Conditioned Dendritic Cells

**Figure 4:**
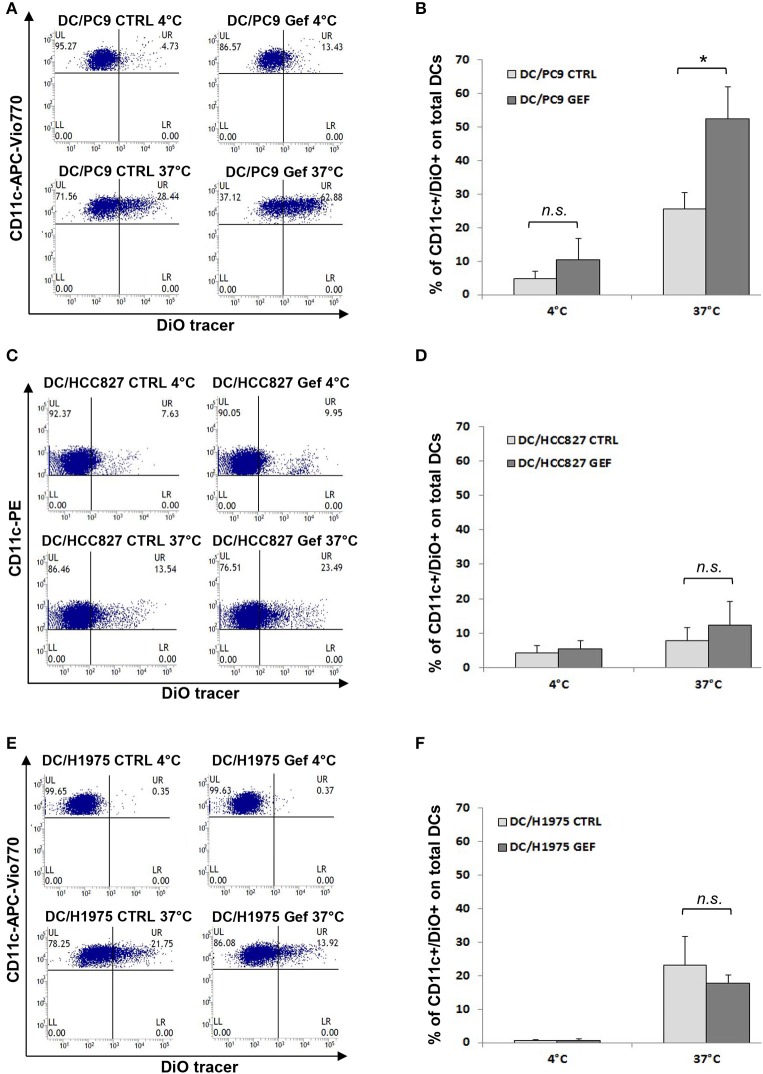
Gefitinib-induced CD47 down-regulation promotes tumor cell phagocytosis by dendritic cells. Representative flow cytometric analyses and mean ± SD (*N* = 4 independent healthy donors) of phagocytic activity of monocyte-derived dendritic cells (see Methods) against PC9 **(A,B)**, HCC827 **(C,D)**, and H1975 cells **(E,F)** treated with DMSO (CTRL) or gefitinib (GEF) as indicated. Cancer cells exposed to the drug for 48 h were labeled with DiO tracer and then co-cultured with dendritic cells for 2 h at a 1:1 ratio. Phagocytosis assays were also run at 4°C as controls. Histograms represent the percentages of positive cells for both CD11c and DiO tracer relative to total dendritic cells (*p < 0.05, n.s., not significant, paired two-tailed Student's *t*-test).

Since the balance between CD47 and ecto-CRT expression determines the susceptibility of cancer cells to engulfment by phagocytes, based on our results (Figures 2C,D), we hypothesized that gefitinib treatment could promote phagocytosis of NSCLC cells by CD47 down-regulation. To test our hypothesis, we performed phagocytosis assay using monocyte-derived dendritic cells from healthy donors and, as target cells, we selected PC9, HCC827, and H1975 cell lines cultured in the presence of gefitinib or DMSO for 48 h. These cell lines were included because of their differential mutational status (EGFR del19 mutation and gefitinib resistant T790M mutation, respectively) and of their differential CD47/ecto-CRT balance following treatment with gefitinib. Cell incubation time was kept at 48 h, since at this time point CD47 down-regulation and ecto-CRT increase were detectable without high levels of apoptosis. Gefitinib-treated and untreated cells were then labeled with the fluorescent tracer DiO (see Methods) and then co-cultured for 2 h with monocyte-derived dendritic cells (29, 40). As shown in Figures 4A,B, PC9 cells treated with gefitinib were engulfed more efficiently than untreated controls, in line with the significant down-regulation of CD47 induced by gefitinib in these cells (Figures 2A,C). Conversely, gefitinib treatment did not significantly affect HCC827 and H1975 cell uptake by dendritic cells (Figures 4C–F). These findings indicate that the marked ecto-CRT up-regulation observed in gefitinib-treated H1975 cells (Figures 2B,D) is not sufficient to increase cell susceptibility to phagocytosis in the absence of a substantial decrease of CD47 (Figures 2A,C).

**Figure 5:**
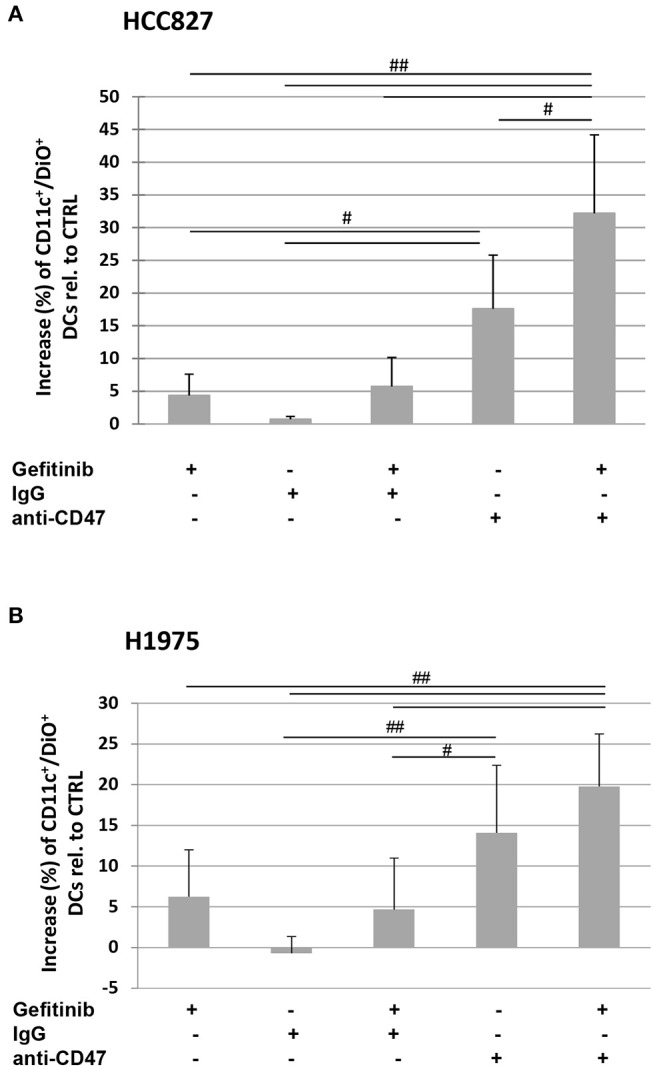
Blocking of CD47 on tumor cells induces phagocytosis by dendritic cells. Dendritic cells were co-cultured with DiO tracer-labeled HCC827 **(A)** and H1975 **(B)** cancer cells in the presence of IgG isotype control or anti-CD47 mAb as indicated. Shown is the mean (± SD, *N* = 3 independent healthy donors) percentage increase of CD11c/DiO tracer double positive cells, relative to dendritic cells co-cultured with DMSO-treated tumor cells (^#^*p* < 0.05, ^##^*p* < 0.01, ANOVA with Fisher's *post hoc* analysis).

Indeed, the addition of a CD47-specific blocking mAb to gefitinib-treated or untreated HCC827 and H1975 cells significantly increased the percentage of DiO-positive dendritic cells, while addition of an isotype control did not affect tumor cell engulfment (Figures 5A,B). This indicates that down-regulation of CD47 in gefitinib-treated NSCLC cells is *per se* sufficient to enhance antitumor immunity by improving cell recognition and engulfment by immune phagocytes.

### CD47 Expression Increases in NSCLC Cells Acquiring Gefitinib Resistance *in vitro*

**Figure 6:**
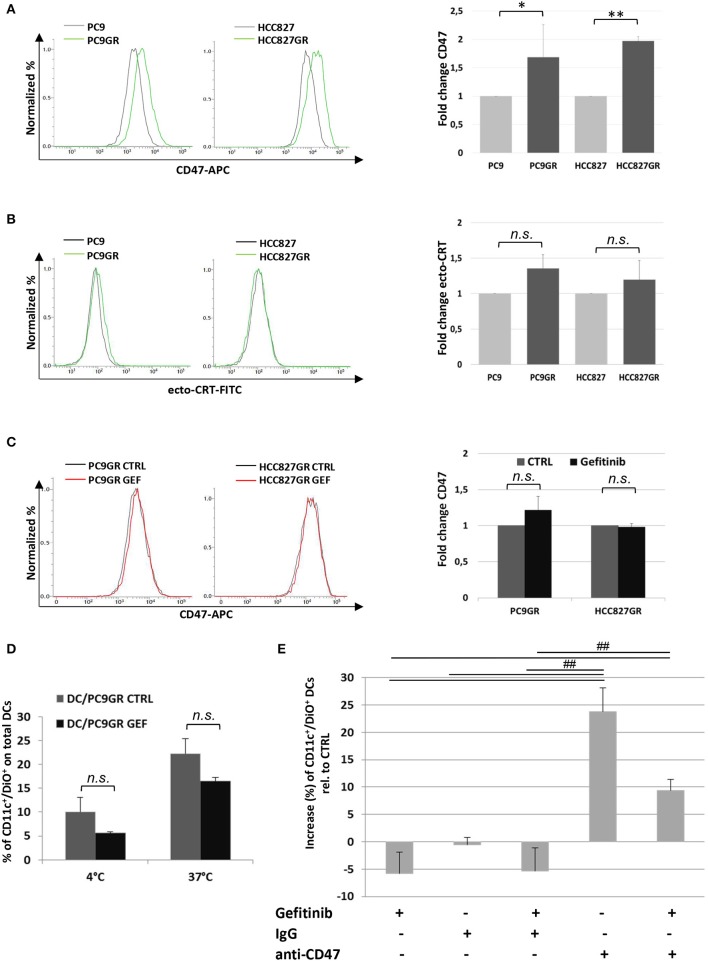
Expression levels of surface CD47 increase in cancer cells acquiring resistance to gefitinib and inhibit tumor cell phagocytosis by dendritic cells. Surface CD47 **(A)** and ecto-CRT expression **(B)** in gefitinib-sensitive PC9 and HCC827 (gray lines) and resistant PC9GR and HCC827GR (green lines) cell lines. Representative flow cytometric histograms (left) and mean (± SD, *N* = 3–5) fold changes of treatment-resistant over sensitive cells (right). **(C)** Representative flow cytometric histogram plots (left) and mean (± SD) fold changes (right) of surface CD47 levels in resistant cell lines treated with DMSO (CTRL) or gefitinib (GEF) as indicated. Acquisition of resistance to gefitinib abolished drug-induced CD47 down-regulation in PC9GR (*p < 0.05, **p < 0.01, n.s., not significant, paired two-tailed Student's *t*-test). **(D)** Mean ± SD (*N* = 3 independent healthy donors) of phagocytic activity of monocyte-derived dendritic cells against PC9GR cells in the absence or presence of gefitinib treatment, performed at 4°C as control and at 37°C. Histograms represent the percentages of positive cells for both CD11c and DiO tracer relative to total dendritic cells (paired two-tailed Student's *t*-test. n.s., not significant). **(E)** Dendritic cells were co-cultured with gefitinib-treated, DiO tracer-labeled PC9GR cells in the presence of IgG isotype control or anti-CD47 mAb. Shown is the mean ± SD (*N* = 3 independent healthy donors) percent change of CD11c^+^/DiO^+^ tracer double positive dendritic cells, relative to dendritic cells co-cultured with DMSO-treated tumor cells (^##^*p* < 0.01, ANOVA with Fisher's *post hoc* analysis).

The development of TKI resistance is the main limiting factor of this otherwise effective target therapy in patients affected by EGFR-mutant lung cancer. Given the increasing evidence of the involvement of tumor microenvironment in remodeling cancer cell responsiveness to TKI therapy, we investigated the expression of CD47 and ecto-CRT in PC9 and HCC827 cell lines after the acquisition of gefitinib resistance *in vitro*. Gefitinib-resistant PC9GR and HCC827GR cells were generated upon exposure to gradually increasing concentrations of the drug. Flow cytometry analyses showed a significant increase of surface CD47 expression in both PC9GR and HCC827GR relative to the parental cell lines (Figure 6A). Conversely, the development of gefitinib resistance was not associated with higher expression of CRT on the plasma membrane (Figure 6B). As expected, treatment of PC9GR and HCC827GR cells with gefitinib for 48 h did not affect the expression levels of CD47 (Figure 6C) and failed to promote tumor cell phagocytosis by dendritic cells (Figure 6D). In contrast, blockade of the CD47/SIRPα axis by the addition of a CD47-specific mAb significantly increased PC9GR phagocytosis by dendritic cells in the presence or absence of gefitinib (Figure 6E).

## Discussion

Our study presents compelling evidence supporting the modulation of CD47 as a novel and important determinant of antitumor immunity in the response to EGFR TKI target therapy. In particular, our findings of overexpressed CD47 in transcriptomic analysis of patients with EGFR-mutated NSCLC, as well as in tumor cell lines acquiring EGFR TKI resistance, identify CD47 as target to be further explored for the immunotherapy treatment of naïve and resistant EGFR-mutant NSCLCs.

The development of resistance occurs consistently in patients affected by EGFR-mutant NSCLCs following first-line treatment with gefitinib and other TKIs. Secondary mutations in the EGFR gene, including T790M, account for more than 50% of resistant cases, followed by the MET gene amplification and the activation of other parallel pathways (41–43). In addition, histological transformation to SCLC may occur in 3–10% of cases (11). Recently, the European Medicines Agency extended the approval of immunotherapy with ICIs to EGFR- and ALK-positive tumors after failure of appropriate targeted therapy. However, the response of these patients to ICI therapy remains poor (13–15), indicating that blocking the PD1/PD-L1 axis is not sufficient to obtain an adequate antitumor immune response.

Several studies documented that first-generation EGFR TKIs improve the interaction between natural killer (NK) and tumor cells favoring immune-mediated cytotoxicity, indicating that EGFR inhibitors can enhance innate tumor immune surveillance (44–46). Despite that, the potential impact of this therapy on immune recognition and elimination of cancer cells by phagocytes remains underexplored. Disruption of the CD47/SIRPα axis with specific mAbs may promote cancer cell elimination by macrophages, and it is a potential immunotherapeutic strategy recently described for different cancers, including SCLC and NSCLC (24, 26, 47–49). Our analysis of large transcriptomic dataset identified higher expression levels of CD47 mRNA, but not of CRT mRNA, in primary lung adenocarcinomas with EGFR mutations as compared to those with different oncogenic mutations. Functional relevance of CD47 overexpression is indicated by our data; although gefitinib down-regulates CD47 and increases ecto-CRT in almost all cell lines tested, it is the decrement of CD47 that results in enhanced phagocytosis of cancer cells by dendritic cells. These results suggest that gefitinib could enhance antitumor immunity by improving lung cancer cell recognition and engulfment by immune phagocytes chiefly through CD47 down-regulation, thereby inhibiting tumor cell viability not only through TK-dependent mechanisms but also by enhancing innate anticancer immune responses.

At variance with previous works using anti-EGFR mAbs 7A7 F(ab′)_2_ in mice (50) and cetuximab in humans (36), in our experimental system, gefitinib does not induce ICD, as it fails to induce established ICD markers (e.g. HMGB1 secretion) in the cells where it promotes widespread apoptosis. Moreover, our results show that the activation of type I IFN pathway is not sufficient to improve the immunogenic features of gefitinib-induced apoptosis. On the other hand, the induction of HSP70 surface exposure and its release by gefitinib-treated tumor cells point to additional immune-modulatory effects of the EGFR TKI.

Our findings that gefitinib-induced down-regulation of CD47 promotes cancer cell phagocytosis in responsive cells and that establishment of gefitinib resistance reverts this response indicate a novel immune mechanism for EGFR TKI therapy, warranting further validation in preclinical and clinical studies. The relevance of CD47 as target in this setting is also underscored by the data obtained in PC9GR cells and in H1975 cells, which harbor a T790M mutation, showing that blocking the CD47/SIRP1α axis promotes cancer cell elimination by dendritic cells also in NSCLC cell lines in which gefitinib has minimal or no effect on CD47 expression. On these bases, evaluation of CD47 expression in patients with EGFR-mutant NSCLCs becoming resistant to target therapy may justify the subsequent adoption of CD47-targeting immunotherapeutic options. In support of this strategy, the administration of CD47-specific mAb inhibited *in vivo* the growth of xenografted tumors developed from patient-derived lung cancer cells or cancer stem cells by recruiting macrophages into the tumor microenvironment (49). Moreover, the administration of CD47-blocking antibodies or targeted inactivation of the *Cd47* gene in humanized mouse models markedly inhibited SCLC tumor growth (24). These evidences could support immunotherapy with CD47-blocking agents as a viable option also in patients undergoing histopathological transformation after EGFR-TKI therapy.

## Conclusions

Several studies conducted over the last few years support an important role of the tumor microenvironment in mutant NSCL adenocarcinoma, especially in TKI resistance development and its dynamic remodeling by target therapy. Increasing awareness of an essential contribution by innate immunity in tumor immune surveillance and in metastasis control is advocated whereby new immunotherapeutic options are becoming available for management of relapsed EGFR mutant patients. In particular, promotion of tumor cells elimination by phagocytosis could be successfully achieved through the administration of anti-CD47 mAbs. The effectiveness of CD47 blockade also following gefitinib treatment, as shown in our experiments *in vitro*, supports the development of therapeutic strategies in which anti-CD47 immunotherapy and target therapy may be combined to minimize the development of resistant clones responsible for tumor relapse.

## Data Availability Statement

The raw data supporting the conclusions of this article will be made available by the authors, without undue reservation, to any qualified researcher.

## Author Contributions

AN performed most of the experiments, analyzed the data, and drafted the figures and parts of the manuscript. LR provided support with the statistical analysis and performed *in silico* analyses. IS, MV, and MC performed some of the experiments and analyzed the data. BM helped set up phagocytosis assays. FS, SP, and MT critically revised the manuscript. CS made significant contributions to data interpretation, manuscript writing, and revision. VC conceived and supervised the study and critically revised the manuscript. JDC conceived the study, developed the experimental design, analyzed and interpreted the data, and wrote the manuscript. All authors contributed toward drafting and revising the paper and approved the final manuscript.

### Conflict of Interest

The authors declare that the research was conducted in the absence of any commercial or financial relationships that could be construed as a potential conflict of interest.

## References

[1] Molina JR Yang P Cassivi SD Schild SE Adjei AA. Non-small cell lung cancer: epidemiology, risk factors, treatment, and survivorship. Mayo Clin Proc. (2008) 83:584–94. 10.4065/83.5.584 18452692 PMC2718421

[2] Bender E. Epidemiology: the dominant malignancy. Nature. (2014) 513:S2–3. 10.1038/513S2a 25208070

[3] Russo A Franchina T Ricciardi GRR Smiroldo V Picciotto M Zanghi M. Third generation EGFR TKIs in EGFR-mutated NSCLC: where are we now and where are we going. Crit Rev Oncol Hematol. (2017) 117:38–47. 10.1016/j.critrevonc.2017.07.003 28807234

[4] Bivona TG Doebele RC. A framework for understanding and targeting residual disease in oncogene-driven solid cancers. Nat Med. (2016) 22:472–8. 10.1038/nm.4091 27149220 PMC5384713

[5] Rotow J Bivona TG. Understanding and targeting resistance mechanisms in NSCLC. Nat Rev Cancer. (2017) 17:637–58. 10.1038/nrc.2017.84 29068003

[6] Im JS Herrmann AC Bernatchez C Haymaker C Molldrem JJ Hong WK. Immune-modulation by epidermal growth factor receptor inhibitors: implication on anti-tumor immunity in lung cancer. PLoS ONE. (2016) 11:e0160004. 10.1371/journal.pone.0160004 27467256 PMC4965069

[7] Yao Z Fenoglio S Gao DC Camiolo M Stiles B Lindsted T. TGF-beta IL-6 axis mediates selective and adaptive mechanisms of resistance to molecular targeted therapy in lung cancer. Proc Natl Acad Sci USA. (2010) 107:15535–40. 10.1073/pnas.1009472107 20713723 PMC2932568

[8] Akbay EA Koyama S Carretero J Altabef A Tchaicha JH Christensen CL. Activation of the PD-1 pathway contributes to immune escape in EGFR-driven lung tumors. Cancer Discov. (2013) 3:1355–63. 10.1158/2159-8290.CD-13-0310 24078774 PMC3864135

[9] Su S Dong ZY Xie Z Yan LX Li YF Su J. Strong programmed death ligand 1 expression predicts poor response and *de novo* resistance to EGFR tyrosine kinase inhibitors among NSCLC patients with EGFR mutation. J Thorac Oncol. (2018) 13:1668–75. 10.1016/j.jtho.2018.07.016 30056164

[10] Choe C Shin YS Kim C Choi SJ Lee J Kim SY. Crosstalk with cancer-associated fibroblasts induces resistance of non-small cell lung cancer cells to epidermal growth factor receptor tyrosine kinase inhibition. Onco Targets Ther. (2015) 8:3665–78. 10.2147/OTT.S89659 26676152 PMC4676617

[11] Remon J Ahn MJ Girard N Johnson M Kim DW Lopes G. Advanced-stage non-small cell lung cancer: advances in thoracic oncology 2018. J Thorac Oncol. (2019) 14:1134–55. 10.1016/j.jtho.2019.03.022 31002952

[12] Tabchi S Kourie HR Kattan J. Adding checkpoint inhibitors to tyrosine kinase inhibitors targeting EGFR/ALK in non-small cell lung cancer: a new therapeutic strategy. Invest New Drugs. (2016) 34:794–6. 10.1007/s10637-016-0383-2 27562868

[13] Garassino MC Cho BC Kim JH Mazieres J Vansteenkiste J Lena H. Durvalumab as third-line or later treatment for advanced non-small-cell lung cancer (ATLANTIC): an open-label, single-arm, phase 2 study. Lancet Oncol. (2018) 19:521–36. 10.1016/S1470-2045(18)30144-X 29545095 PMC7771363

[14] Garassino MC Gelibter AJ Grossi F Chiari R Soto Parra H Cascinu S. Italian nivolumab expanded access program in nonsquamous non-small cell lung cancer patients: results in never-smokers and EGFR-mutant patients. J Thorac Oncol. (2018) 13:1146–55. 10.1016/j.jtho.2018.04.025 29730379

[15] Mazieres J Drilon A Lusque A Mhanna L Cortot AB Mezquita L. Immune checkpoint inhibitors for patients with advanced lung cancer and oncogenic driver alterations: results from the IMMUNOTARGET registry. Ann Oncol. (2019) 30:1321–8. 10.1093/annonc/mdz167 31125062 PMC7389252

[16] Cho JH Jung HA Lee SH Ahn JS Ahn MJ Park K. Impact of EGFR mutation on the clinical efficacy of PD-1 inhibitors in patients with pulmonary adenocarcinoma. J Cancer Res Clin Oncol. (2019) 145:1341–9. 10.1007/s00432-019-02889-0 30900155 PMC11810305

[17] Barclay AN Van den Berg TK. The interaction between signal regulatory protein alpha (SIRPalpha) and CD47: structure, function, and therapeutic target. Annu Rev Immunol. (2014) 32:25–50. 10.1146/annurev-immunol-032713-120142 24215318

[18] Brown EJ Frazier WA. Integrin-associated protein (CD47) and its ligands. Trends Cell Biol. (2001) 11:130–5. 10.1016/S0962-8924(00)01906-1 11306274

[19] Chao MP Weissman IL Majeti R. The CD47-SIRPalpha pathway in cancer immune evasion and potential therapeutic implications. Curr Opin Immunol. (2012) 24:225–32. 10.1016/j.coi.2012.01.010 22310103 PMC3319521

[20] Oldenborg PA Zheleznyak A Fang YF Lagenaur CF Gresham HD Lindberg FP. Role of CD47 as a marker of self on red blood cells. Science. (2000) 288:2051–4. 10.1126/science.288.5473.2051 10856220

[21] Jaiswal S Jamieson CH Pang WW Park CY Chao MP Majeti R. CD47 is upregulated on circulating hematopoietic stem cells and leukemia cells to avoid phagocytosis. Cell. (2009) 138:271–85. 10.1016/j.cell.2009.05.046 19632178 PMC2775564

[22] Willingham SB Volkmer JP Gentles AJ Sahoo D Dalerba P Mitra SS. The CD47-signal regulatory protein alpha (SIRPa) interaction is a therapeutic target for human solid tumors. Proc Natl Acad Sci USA. (2012) 109:6662–7. 10.1073/pnas.1121623109 22451913 PMC3340046

[23] Uluckan O Becker SN Deng H Zou W Prior JL Piwnica-Worms D. CD47 regulates bone mass and tumor metastasis to bone. Cancer Res. (2009) 69:3196–204. 10.1158/0008-5472.CAN-08-3358 19276363 PMC2763641

[24] Weiskopf K Jahchan NS Schnorr PJ Cristea S Ring AM Maute RL. CD47-blocking immunotherapies stimulate macrophage-mediated destruction of small-cell lung cancer. J Clin Invest. (2016) 126:2610–20. 10.1172/JCI81603 27294525 PMC4922696

[25] Zhao H Wang J Kong X Li E Liu Y Du X. CD47 Promotes tumor invasion and metastasis in non-small cell lung cancer. Sci Rep. (2016) 6:29719. 10.1038/srep29719 27411490 PMC4944213

[26] Zhang X Fan J Wang S Li Y Wang Y Li S. Targeting CD47 and autophagy elicited enhanced antitumor effects in non-small cell lung cancer. Cancer Immunol Res. (2017) 5:363–75. 10.1158/2326-6066.CIR-16-0398 28351890

[27] Kong F Gao F Li H Liu H Zhang Y Zheng R. CD47: a potential immunotherapy target for eliminating cancer cells. Clin Transl Oncol. (2016) 18:1051–5. 10.1007/s12094-016-1489-x 26830085

[28] Weiskopf K. Cancer immunotherapy targeting the CD47/SIRPalpha axis. Eur J Cancer. (2017) 76:100–9. 10.1016/j.ejca.2017.02.013 28286286

[29] Montico B Lapenta C Ravo M Martorelli D Muraro E Zeng B. Exploiting a new strategy to induce immunogenic cell death to improve dendritic cell-based vaccines for lymphoma immunotherapy. Oncoimmunology. (2017) 6:e1356964. 10.1080/2162402X.2017.1356964 29147614 PMC5674955

[30] Obeid M Tesniere A Panaretakis T Tufi R Joza N van Endert P. Ecto-calreticulin in immunogenic chemotherapy. Immunol Rev. (2007) 220:22–34. 10.1111/j.1600-065X.2007.00567.x 17979837

[31] Fucikova J Becht E Iribarren K Goc J Remark R Damotte D. Calreticulin expression in human non-small cell lung cancers correlates with increased accumulation of antitumor immune cells and favorable prognosis. Cancer Res. (2016) 76:1746–56. 10.1158/0008-5472.CAN-15-1142 26842877

[32] Santini SM Lapenta C Santodonato L D'Agostino G Belardelli F Ferrantini M IFN-alpha in the generation of dendritic cells for cancer immunotherapy. Handb Exp Pharmacol. (2009) 188:295–317. 10.1007/978-3-540-71029-5_14 19031032

[33] Okayama H Kohno T Ishii Y Shimada Y Shiraishi K Iwakawa R. Identification of genes upregulated in ALK-positive and EGFR/KRAS/ALK-negative lung adenocarcinomas. Cancer Res. (2012) 72:100–11. 10.1158/0008-5472.CAN-11-1403 22080568

[34] Yamauchi M Yamaguchi R Nakata A Kohno T Nagasaki M Shimamura T. Epidermal growth factor receptor tyrosine kinase defines critical prognostic genes of stage I lung adenocarcinoma. PLoS ONE. (2012) 7:e43923. 10.1371/journal.pone.0043923 23028479 PMC3446964

[35] Li Q Birkbak NJ Gyorffy B Szallasi Z Eklund AC. Jetset: selecting the optimal microarray probe set to represent a gene. BMC Bioinformatics. (2011) 12:474. 10.1186/1471-2105-12-474 22172014 PMC3266307

[36] Pozzi C Cuomo A Spadoni I Magni E Silvola A Conte A. The EGFR-specific antibody cetuximab combined with chemotherapy triggers immunogenic cell death. Nat Med. (2016) 22:624–31. 10.1038/nm.4078 27135741

[37] Kepp O Senovilla L Vitale I Vacchelli E Adjemian S Agostinis P. Consensus guidelines for the detection of immunogenic cell death. Oncoimmunology. (2014) 3:e955691. 10.4161/21624011.2014.955691 25941621 PMC4292729

[38] Liu P Zhao L Pol J Levesque S Petrazzuolo A Pfirschke C. Author correction: crizotinib-induced immunogenic cell death in non-small cell lung cancer. Nat Commun. (2019) 10:1883. 10.1038/s41467-019-09838-y 30996258 PMC6470160

[39] Sistigu A Yamazaki T Vacchelli E Chaba K Enot DP Adam J. Cancer cell-autonomous contribution of type I interferon signaling to the efficacy of chemotherapy. Nat Med. (2014) 20:1301–9. 10.1038/nm.3708 25344738

[40] Cox MC Castiello L Mattei M Santodonato L D'Agostino G Muraro E. Clinical and antitumor immune responses in relapsed/refractory follicular lymphoma patients after intranodal injections of IFNα-dendritic cells and Rituximab: a phase I clinical trial. Clin Cancer Res. (2019) 25:5231–41. 10.1158/1078-0432.CCR-19-0709 31171545

[41] Yu HA Arcila ME Rekhtman N Sima CS Zakowski MF Pao W. Analysis of tumor specimens at the time of acquired resistance to EGFR-TKI therapy in 155 patients with EGFR-mutant lung cancers. Clin Cancer Res. (2013) 19:2240–7. 10.1158/1078-0432.CCR-12-2246 23470965 PMC3630270

[42] Lee CK Kim S Lee JS Lee JE Kim SM Yang IS. Next-generation sequencing reveals novel resistance mechanisms and molecular heterogeneity in EGFR-mutant non-small cell lung cancer with acquired resistance to EGFR-TKIs. Lung Cancer. (2017) 113:106–14. 10.1016/j.lungcan.2017.09.005 29110836

[43] Kosaka T Yatabe Y Endoh H Yoshida K Hida T Tsuboi M. Analysis of epidermal growth factor receptor gene mutation in patients with non-small cell lung cancer and acquired resistance to gefitinib. Clin Cancer Res. (2006) 12:5764–9. 10.1158/1078-0432.CCR-06-0714 17020982

[44] Dominguez C Tsang KY Palena C. Short-term EGFR blockade enhances immune-mediated cytotoxicity of EGFR mutant lung cancer cells: rationale for combination therapies. Cell Death Dis. (2016) 7:e2380. 10.1038/cddis.2016.297 27685624 PMC5059888

[45] He S Yin T Li D Gao X Wan Y Ma X. Enhanced interaction between natural killer cells and lung cancer cells: involvement in gefitinib-mediated immunoregulation. J Transl Med. (2013) 11:186. 10.1186/1479-5876-11-186 23937717 PMC3766712

[46] Kim H Kim SH Kim MJ Kim SJ Park SJ Chung JS. EGFR inhibitors enhanced the susceptibility to NK cell-mediated lysis of lung cancer cells. J Immunother. (2011) 34:372–81. 10.1097/CJI.0b013e31821b724a 21499124

[47] Feng M Jiang W Kim BYS Zhang CC Fu YX Weissman IL. Phagocytosis checkpoints as new targets for cancer immunotherapy. Nat Rev Cancer. (2019) 19:568–86. 10.1038/s41568-019-0183-z 31462760 PMC7002027

[48] Orozco-Morales M Soca-Chafre G Barrios-Bernal P Hernandez-Pedro N Arrieta O. Interplay between cellular and molecular inflammatory mediators in lung cancer. Mediators Inflamm. (2016) 2016:3494608. 10.1155/2016/3494608 26941482 PMC4749813

[49] Liu L Zhang L Yang L Li H Li R Yu J. Anti-CD47 Antibody as a targeted therapeutic agent for human lung cancer and cancer stem cells. Front Immunol. (2017) 8:404. 10.3389/fimmu.2017.00404 28484448 PMC5399041

[50] Garrido G Rabasa A Sanchez B Lopez MV Blanco R Lopez A. Induction of immunogenic apoptosis by blockade of epidermal growth factor receptor activation with a specific antibody. J Immunol. (2011) 187:4954–66. 10.4049/jimmunol.1003477 21984704

