# Supplementary figures and images for: Enhanced Expression of CD47 Is Associated With Off-Target Resistance to Tyrosine Kinase Inhibitor Gefitinib in NSCLC
Source: Front Immunol. 2020 Jan 31;10:3135. doi: 10.3389/fimmu.2019.03135 (PMC7004973; doi:10.3389/fimmu.2019.03135)

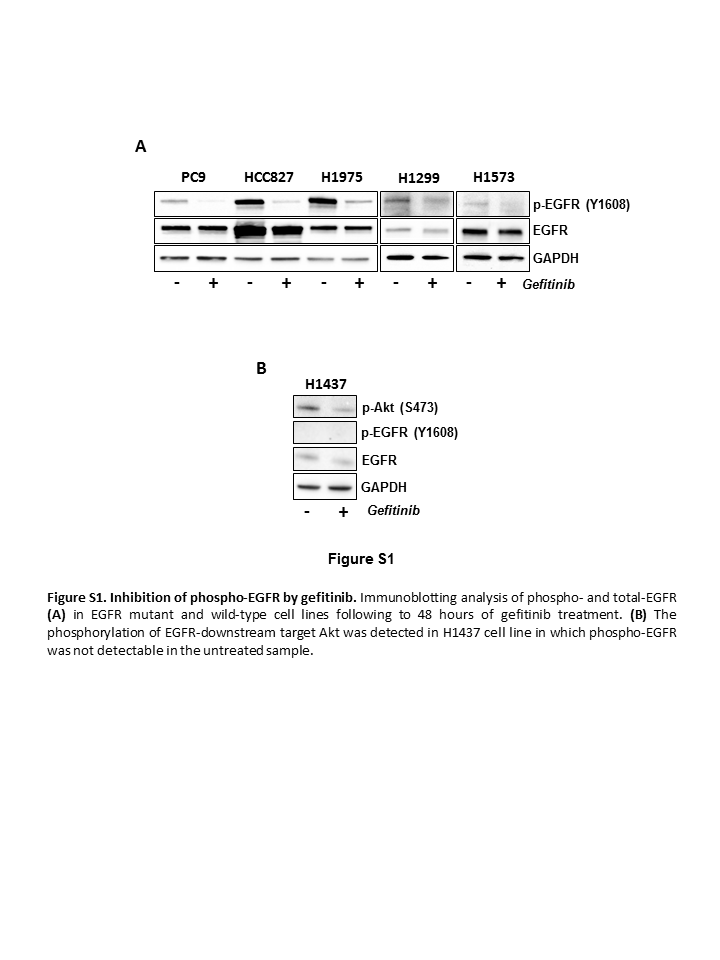

Supplement: Supplementary file 1 [file Image_1.TIF]

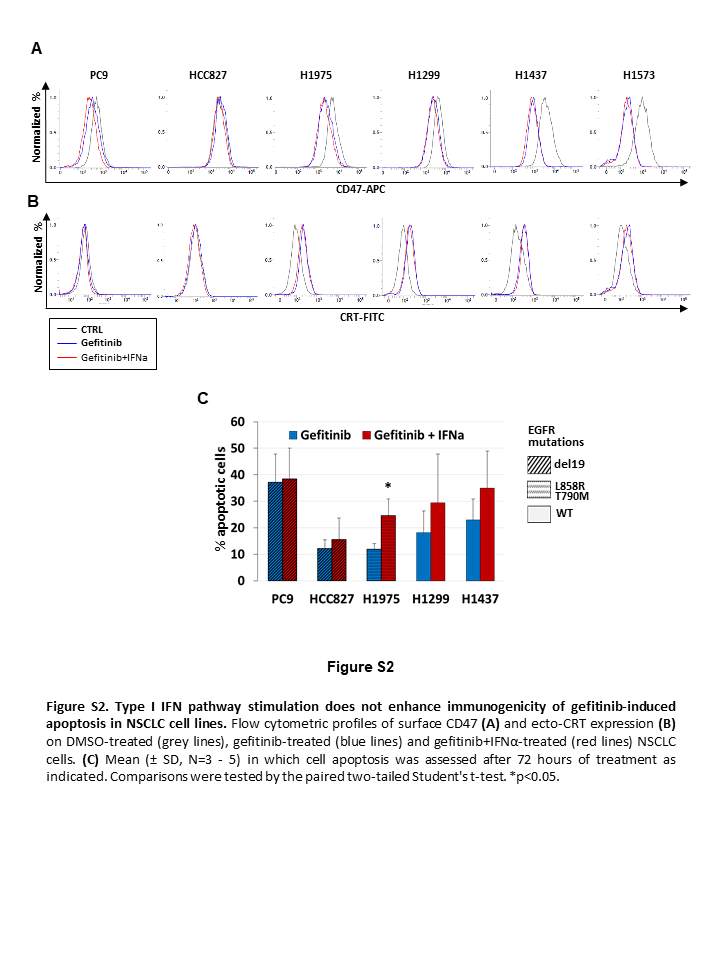

Supplement: Supplementary file 2 [file Image_2.tif]
